# Simulating Speech Error Patterns Across Languages and Different Datasets

**DOI:** 10.1177/0023830920987268

**Published:** 2021-02-26

**Authors:** Sofia Strömbergsson, Jana Götze, Jens Edlund, Kristina Nilsson Björkenstam

**Affiliations:** Department of Clinical Science, Intervention and Technology (CLINTEC), Karolinska Institutet (KI), Sweden; Department of Speech, Music and Hearing, KTH Royal Institute of Technology, Sweden; Department of Linguistics, Stockholm Universitet, Sweden

**Keywords:** Phonological acquisition, corpus linguistics, phonological typology, speech sound disorders

## Abstract

Children’s speech acquisition is influenced by universal and language-specific forces. Some speech error patterns (or phonological processes) in children’s speech are observed in many languages, but the same error pattern may have different effects in different languages. We aimed to explore phonological effects of the same speech error patterns across different languages, target audiences and discourse modes, using a novel method for large-scale corpus investigation. As an additional aim, we investigated the face validity of five different phonological effect measures by relating them to subjective ratings of assumed effects on intelligibility, as provided by practicing speech-language pathologists. Six frequently attested speech error patterns were simulated in authentic corpus data: backing, fronting, stopping, /r/-weakening, cluster reduction and weak syllable deletion—each simulation resulting in a “misarticulated” version of the original corpus. Phonological effects were quantified using five separate metrics of phonological complexity and distance from expected target forms. Using Swedish child-speech data as a reference, phonological effects were compared between this reference and a) child speech in Norwegian and English, and b) data representing different modes of discourse (spoken/written) and target audiences (adults/children) in Swedish. Of the speech error patterns, backing—the one atypical pattern of those included—was found to cause the most detrimental effects, across languages as well as across modes and speaker ages. However, none of the measures reflects intuitive rankings as provided by clinicians regarding effects on intelligibility, thus corroborating earlier reports that phonological competence is not translatable into levels of intelligibility.

## 1 Introduction

One important driver of children’s speech and language acquisition is their advancing cognitive and motor capacities. And as children’s physiological development is largely universal, some developmental trends are found regardless of what language the child is acquiring. For example, children typically acquire plosives before fricatives, and labial and coronal consonants before velar consonants (Vihman, 1993; Lee et al., 2010; Davis & MacNeilage, 1995; McLeod & Crowe, 2018). Universal trends are also observed in children’s delayed phonological acquisition, such that the same speech error patterns (or phonological processes)^1^ are observed across many languages. For example, velar fronting has been reported in languages like English, Swedish, and Cantonese (e.g., Cleland et al., 2017; Strömbergsson et al., 2015; Cheung & Abberton, 2000). On the other hand, the words and sounds that children hear drive their developmental trajectory in the direction of the surrounding language (e.g., Gierut & Dale, 2007; Yavaş, 2014). Such language-dependent pressure explains why the voiced fricative /v/ is acquired earlier in Swedish, Estonian and Bulgarian than in English (Ingram, 1988), and why English-learning children’s first words are predominantly monosyllabic, whereas same-aged children acquiring Japanese, Swedish and French produce more disyllabic or longer words (Vihman, 1993). In descriptions of children’s speech error patterns across languages, researchers point to a shared influence of universal and language-dependent forces (e.g., Clausen & Fox-Boyer, 2017; Hua & Dodd, 2006). The present study targets a related question that also resides in the intersection between language-universal and language-dependent pressure, namely: can typological differences between languages result in the same error pattern having more detrimental effects in one language than in another?

Much research underlying the identification of universal and language-specific trends in children’s speech and language acquisition has been based on distributional characteristics of adult language, for example, in the computing of probabilistic phonotactics (Stokes, 2014; Storkel, 2004). Although reports have shown that certain aspects of word frequency that influence children’s phonological acquisition are insensitive to whether the analysis is based on adult or child data (Gierut & Dale, 2007), others have identified distributional differences in child data compared to adult data (Dollaghan, 1994; Strömbergsson et al., 2017), raising concerns that more ecologically valid sources should be preferred. The lack of valid sources is a problem which is being remediated through the steadily increasing availability of both child-directed and child-produced speech data, for example, through initiatives like CHILDES (MacWhinney, 2000), PhonBank (Rose & MacWhinney, 2014) and LENA (Ganek & Eriks-Brophy, 2018). However, until such resources are universally available, many researchers have to rely on suboptimal resources. Thus, the need for estimating the degree of uncertainty in predictions of children’s spoken language based on adult language use still remains.

We investigate phonological effects of six frequently observed speech error patterns through the simulation of these patterns in language data collected from Swedish, Norwegian, and English sources in order to glean how effects of the same error pattern vary across the three languages. Furthermore, by comparing these effects on different data subsets—written as well as spoken data, and data produced by adults as well as by children—we seek to explore the viability of basing investigations of children’s phonological acquisition on more readily available sources than the ideal and most ecologically valid ones.

### 1.1 Quantifying phonological complexity and accuracy

Children’s emerging phonological systems can be described from different perspectives. On the one hand, phonological characteristics of observed speech production can be expressed in structural terms, independently of assumed target forms. Such independent aspects of children’s speech production can be quantified as measures of phonological complexity and are particularly useful in the description of children’s speech production at early stages of acquisition (Stoel-Gammon, 2010). The Index of Phonetic Complexity (IPC) (Jakielski, 1998; Jakielski, 2016) is one such measure. This index is based on early work by Davis and MacNeilage (1995), describing how children’s babbling behavior and physiological restrictions shape their early speech production. The IPC takes into account the type of sound segments produced, as well as how these are combined; sounds that are typically acquired later receive higher scores than those acquired earlier, and combinations of sounds that are more complex (e.g., heterorganic clusters) receive higher scores than those that are less articulatorily demanding. The IPC has been used in studies of the interplay between phonetic complexity and stuttering, in children, teenagers, and adults speaking English (Howell et al., 2006) and Spanish (Howell & Au-Yeung, 2007). Another independent measure of phonological complexity is the Word Complexity Measure (WCM) (Stoel-Gammon, 2010). Just like the IPC, the WCM codes later acquired sounds and sound combinations as more complex than those acquired earlier. Unlike the IPC, however, the WCM is sensitive to prosodic aspects like lexical stress and accent. For example, productions with stress on any syllable but the first are regarded as more complex than productions with stress on the first syllable. The WCM measure was originally developed for children acquiring English, but has later been adopted to other languages, for example to Swedish (Marklund et al., 2018). As children’s early speech production—and error patterns in delayed or disordered speech—often involve reduced articulatory effort compared to expected target production (see e.g., Ball, 2015, pp. 103-105), more severe misarticulation can be expected to correspond to greater reduction in phonetic complexity.

In contrast to independent structural measures, relational measures describe the difference between the expected (adult-like) target and the observed (child-produced) version of this target. In the study of children’s phonological acquisition, and especially in the field of atypical development, the Percentage of Consonants Correct (PCC) (Shriberg & Kwiatkowski, 1982) is a well-spread measure for quantifying speech accuracy. In its original version, the PCC is insensitive to the phonetic degree of deviance from target, treating all consonant mismatches alike. Although many variants of the PCC have been suggested to address this limitation, the original version is still used by many as the standard measure of speech accuracy (McLeod & Goldstein, 2012). A more sensitive measure has been suggested by Preston and colleagues, namely the Weighted Speech Sound Accuracy (WSSA) (Preston et al., 2011). The WSSA weighting is motivated to a large extent on assumed effects on intelligibility, giving more penalty to infrequent errors, assumed to have larger detrimental impact on intelligibility (Preston et al., 2011). Based on this reasoning, an error pattern like backing (the substitution of velar [k, g, ŋ] for alveolar correspondences /t, d, n/) is penalized for being an atypical pattern (Dodd, 2005; Hodson & Paden, 1983). Although the WSSA has several desirable features of a speech accuracy measure with potentially high functional relevance, its usefulness in cross-linguistic investigations remains limited, as it has not been developed for other languages than English.

Notably, the general task of quantifying the distance (or similarity) between one sequence of symbols and another is by no means unique to the field of children’s speech production. In applications like recognition of misspellings or alignment of transcriptions, this task is sometimes referred to as pairwise string alignment (PSA). The most frequently used PSA measure is probably the Levenshtein distance measure (Wieling et al., 2009). The Levenshtein distance between two words (i.e., two sequences of characters) is the minimum number of single-character insertions, deletions or substitutions needed to change one word into the other. The Levenshtein distance has been used to quantify the difference between pairs of words produced by speakers of different dialects (Gooskens & Heeringa, 2004). When used in this context, the Levenshtein distance has been found to correlate with objective mutual functional intelligibility scores of closely related languages like Danish, Norwegian, and Swedish (Gooskens, 2006). Hence, the measure can be expected to be revealing aspects of the functional consequences of speech not matching expected target forms.

Among measures quantifying speech production proficiency are also suggestions that combine independent and relational aspects. One such measure is the Phonological Mean Length of Utterance (pMLU) (Ingram, 2002). The pMLU is computed as the segmental length of the child’s word productions plus the number of correct consonants in these productions, divided by the total number of word tokens. Therefore, in addition to being sensitive to the accuracy of the segments produced (just like the PCC), the pMLU also reflects variation in word length (i.e., structural characteristics). However, the pMLU measure yields similar scores for short words produced accurately as for longer words produced less accurately. Hence, the complexity of the intended word targets is unaccounted for by the pMLU. Addressing this limitation, Ingram (2002) suggested the Proportion of Whole-word Proximity (PWP) (Ingram, 2002) as a measure reflecting the complexity of the child’s production in relation to that of the attempted target. The PWP is calculated by dividing the pMLU of the child’s production by the pMLU of the target form. For example, for the production /nana/ for “banana,” the PWP would be 6/9 = .67, whereas the PWP of /nana/ for “nanny” would be 6/6 = 1.0. Of Ingram’s (2002) suggested whole-word measures, the pMLU and the PWP are probably the most widely used (see e.g., Arias and Lleó, 2014). Further, both measures have proved to be sensitive to children’s phonological development over time across different languages (Ingram, 2002; Saaristo-Helin, 2009). However, caution has been raised that language-specific adjustments may be needed when using the pMLU in cross-linguistic comparisons (Saaristo-Helin et al., 2006). This fact, together with the suggestion of the PWP as at least an indirect measure of intelligibility (Ingram, 2002) makes the PWP particularly relevant for inclusion in the present work.

As is clear from the above, many suggestions for quantifying the quality of children’s speech production have been put forth. While some have posited at least an indirect link between speech accuracy and intelligibility (Ingram, 2002; McLeod et al., 2012), the functional validity of different quantifications, that is, to what extent a quantitative accuracy score relates to effects on functional communication, has rarely been tested (Mason et al., 2015). Alternative composite measures are rare, even though many have asserted that a comprehensive measure of phonological acquisition needs to be sensitive to more aspects than to the accuracy of phonological segments (e.g., Ingram, 2002; Mason et al., 2015; Masso et al., 2017). Examination of the relation between different existing measures is necessary when inventorying what measure best reflects the functional consequences of a speech disorder.

### 1.2 Ranking speech error patterns by severity

Intelligible speech is typically a long-term goal in the intervention of speech disorders (Lousada et al., 2014). Hence, although there are multiple considerations weighing into clinical decisions regarding what speech error patterns to prioritize in intervention (see e.g., Rvachew & Nowak, 2001), the ranking of error patterns by their impact on intelligibility would provide additional guidance in the prioritization of treatment goals. Efforts have been made to systematically rank error patterns by (un)intelligibility, but suggested rankings have often been based on clinical intuition, rather than on empirical evidence (Klein & Flint, 2006). For example, it has been suggested that deviant production of speech sounds occurring frequently in the child’s language has more pervasive effects on intelligibility than misarticulation of sounds occurring less frequently (Brown, 1988), that misarticulation involving the neutralization of phonological contrasts are more damaging than those not causing lexical confusion (Ingram, 1989), and that inconsistent misarticulation is more detrimental to intelligibility than consistent misarticulation (Yavaş & Lamprecht, 1988). Of the empirically based research on the ranking of error patterns with regards to their impact on intelligibility, Hodson and Paden’s work (1981, 1983) is probably the most well-known. By observing differences in the distribution of error patterns across English-speaking children grouped by level of intelligibility, Hodson and Paden concluded, for example, that omissions of speech sounds are more detrimental to intelligibility than phonetic distortions, and that atypical error patterns are more disruptive than those often occurring early in typical phonological acquisition (Hodson & Paden, 1981). As such, these observations have described correlation, that is, indirect links, between error patterns and (un)intelligibility. Approaching the issue from a different angle, Klein and Flint (2006) have reported a unique study of more direct links between speech error patterns and effects on intelligibility. Through the simulation of error patterns, these researchers were able to control both the types and the distribution of misarticulation, and, via calculating the proportions of words understood by listeners, to explore their consequences in terms of intelligibility. By ranking the three included speech error patterns—final consonant deletion, stopping and velar fronting—by their impact on intelligibility, final consonant deletion was found to have the most detrimental effect (Klein & Flint, 2006). However, the study has some methodological shortcomings, particularly pertaining to ecological validity. Considering that the results are based on “distorted” phonemic transcriptions being read aloud by an adult male, that is, recordings of an adult speaker producing error patterns that are highly unexpected in adult speech, it cannot be ruled out that listener responses reflect other factors than merely intelligibility. Nevertheless, with the desirable feature of allowing control over the distribution of error patterns, distorting phonemic transcriptions may still be a viable approach to studying the impact of different error patterns in context, particularly for large-scale transcription-based studies.

### 1.3 Phonological structure across different languages

Cross-linguistic explorations of phonological structure need to rely on the phoneme as the unit of analysis, rather than the phone or allophone (McLeod & Crowe, 2018). Further, consonants—rather than vowels, tones or prosodic features—are most often of particular interest in the study of phonological acquisition, within and across languages (McLeod & Crowe, 2018). Inevitably, then, reduction of phonetic detail in children’s actual speech production is often accepted, and a segmental focus assumed, in order for more general patterns of similarities and differences between languages to be revealed.

Historically, the study on speech acquisition and on speech sound disorders in children has predominantly been based on English. However, more recent cross-linguistic research has shed light on phenomena described for English that do not generalize to other languages, due to differences in linguistic structure (Saaristo-Helin et al., 2006; Saaristo-Helin, 2009; Bernhardt et al., 2017; Hua & Dodd, 2006b). Although sharing the same Germanic origin, Swedish and Norwegian differ phonologically from English in a number of ways, see Table 1. For example, the Scandinavian languages have more vowels than English, and tonal accents.

**Table 1.** Phonological descriptions of Swedish, Norwegian, and English. The description of Swedish is based on Riad (2014) and Engstrand (2004), the description of Norwegian on Kristoffersen (2000), and the description of English on Smit (2004).

	Swedish	Norwegian	English
Consonants	18 consonants: /p, b, t, d, k, ɡ, f, v, s, ʂ, ɕ, ʝ, h, m, n, ŋ, l, r/^1^	24 consonants: /p, b, t, d, ʈ, ɖ, k, ɡ, m, n, ɳ, ŋ, ɾ, ɽ, f, s, ʂ, ç, h, ʋ, j, l, ɭ, w/	24 consonants: /p, b, t, d, k, ɡ, m, n, ŋ, f, v, s, z, θ, ð, ʃ, ʒ, h, ʧ, ʤ, j, w, r, l/
Consonant clusters	CC and CCC clusters in onset and coda position. CCCC and CCCCC also occur in final position in inflected forms	CC and CCC clusters in onset and coda position. CCCC also occur in final position in inflected forms	CC and CCC clusters in onset and coda position. CCCC final clusters occur
Vowels and diphthongs	17 vowels: /i:, ɪ, y:, ʏ, ʉ̟, ɵ, e:, ø:, œ, ɛ:, ɛ, u:, ʊ, o:, ɔ, ɑ:, a/	18 vowels: /iː, i, yː, y, ʉː, ʉ, eː, e, øː, ø, æː, æ, uː, u, oː, o, ɑː, ɑ/	14 vowels: /i, ɪ, e, ɛ, æ, ə, ɚ, ɝ, u, ʊ, o, ʌ, ɔ, ɑ/
Diphthongs	-^2^	/æj, øj, æw/ Other diphthongs occur marginally.	/aɪ, aʊ, ɔɪ/
Tonal accents	Accent 1 and Accent 2^3^	Accent 1 and Accent 2	-
Syllable shape	C_(0-3)_VC_(0-5)_	C_(0-3)_VC_(0-4)_	C_(0-3)_VC_(0-4)_
Word shape	Productive concatenative compounding; compounds are one word units, for example, *vattenflaska* (Eng. *water bottle*)	Productive concatenative compounding; compounds are one word units, for example, *vannflaske* (Eng. *water bottle*)	Compounds typically consist of a group of separate words, for example, *water bottle*
Word-level stress	Primary, secondary or no stress	Primary, secondary or no stress	Primary, secondary or no stress

1 The retroflex consonants /ʈ, ɖ, ɳ, ɭ/ are treated as having phonemic status in Swedish by some, see for example, Lundeborg Hammarström (2018). With this view, the Swedish and Norwegian consonant systems are more similar than indicated in the table.

2 Diphthongs are often not considered phonemic in Swedish.

3 Tonal accents are indicated in phonemic transcriptions for Swedish and Norwegian before the primary stressed syllable, with /^1^/ representing accent 1, and /^2^/ representing accent 2. In English phonemic transcriptions, where no tonal accent distinction exists, primary stress is indicated with /ˈ/.

We are not aware of any previous investigations involving large-scale transcription-based quantifications of the phonological effects of simulating common error patterns in authentic linguistic data. Hence, the present investigation constitutes a first effort at this task. Basing the investigation on phonologically transcribed data allows relatively convenient upscaling and extension to other languages. Here, phonological effects of the same error patterns are explored across child-speech transcripts in English, Swedish, and Norwegian. Knowing that Swedish and Norwegian are more similar to one another than either of the two is to English, larger differences are expected between English and the two Scandinavian languages, than between the two Scandinavian languages.

### 1.4 Phonological structure across different linguistic sources

Although ecologically valid linguistic speech data is to be preferred, the reality is that at present both the quantity and the quality of available child-speech corpora differ greatly between languages. Therefore, researchers may have to rely on other types of more readily available data. While some phonological phenomena may be insensitive to the specific mode and target age group of the underlying linguistic data, other factors are less robust. For example, in their investigation of the potential influence of word frequency on phonological acquisition in children with a phonological disorder, Gierut and Dale (2007) demonstrated results that were consistent across both spoken and written data, and across child and adult data, and they concluded, therefore, that for this specific purpose, it was not necessary to rely on a corpus of transcripts of utterances produced by children. However, more recent studies highlight distributional differences across modes of discourse and populations, thus calling for corpus-based language acquisition studies to be based on data collected from more ecologically valid sources, such as children’s lexicons derived from spontaneous speech data (e.g., Daland, 2013; Tsuji et al., 2014). This caution is resonated by Stoel-Gammon (2011), who calls for the study of word frequency effects to include a variety of measures, such as adult word counts, word counts of child input and output, collected from many children, as well as of word input and output in individual children. A systematic investigation of variation in linguistic structure across different modes (spoken vs. written) and different ages (children vs. adults) requires data from four types of sources: child and adult speech data, and texts written for child and adult target audiences. To that end, we explore phonological effects of the same error patterns across such linguistic sources.

### 1.5 Aim

The aim of the current investigation was to explore phonological effects of the same speech error patterns across different languages, target audiences and discourse modes, using a novel method for large-scale corpus investigation. Using phonemic transcripts derived from a dataset of spoken data produced by Swedish children as a reference, and through comparison between this reference and a) corpus data representing other languages (Norwegian and English), and b) Swedish corpus data in different modes of discourse (spoken/written) and intended audiences (adults/children), we aim to explore the following research question:

*How are commonly observed speech error patterns quantitatively ranked by severity across Swedish, Norwegian, and English?*

We quantify severity by measures of phonological complexity and accuracy. Speech error patterns reported as being more severe (e.g., atypical patterns > typical patterns; omissions > substitutions) are also expected to be quantitatively ranked as more severe—across all three languages. We explore this question on the most ecologically valid data, that is, child-produced speech.

Due to the relative scarcity of optimal data sources, the question will also be investigated using less ideal (but more easily accessible) data, namely, text. Hence, as a secondary purpose, we aim to explore the potential distortion of basing investigations of children’s phonological acquisition on material other than that of spoken utterances produced by children, with the following research question:

*Within the same language, how does severity vary across a) the age of the producer (adult vs. child), b) the age of the intended audience (adults vs. children), and c) the mode of linguistic data (spoken vs. written)?*

Based on preliminary data presented in Strömbergsson et al. (2017), where differences in phonotactic distributions were identified between different types of linguistic data within one and the same language, differences are expected also in what effects simulated error patterns may have. With fewer opportunities where an error pattern is applicable, the smaller the expected effect. For example, if the consonant cluster /str/ occurs less frequently in child-produced speech than in other data sources, the effect of cluster reduction can be expected to be less severe in this dataset.

Because the included quantitative measures of severity concern different phonological aspects of the data, and because it is unknown to what extent these aspects relate to consequences in functional communication (e.g., in terms of intelligibility), the face validity of the rankings provided by the included measures is explored through the following research question:

*To what extent do the Swedish estimated rankings of severity correspond to intuitive rankings collected from practicing clinicians?*

There is little empirical evidence of correlations between level of severity and effects on intelligibility. A finding that any of the included measure correlates with clinically intuitive rankings would strengthen the functional validity of this measure.

## 2 Method

### 2.1 Materials

For each of the three languages in this study, a collection of orthographically transcribed spoken data produced by children was selected from open data resources (see Table 2). For child-produced speech, there are high quality corpora for both British and American English. We decided to use both American and British data, which is not unusual in studies of phonological acquisition, for example, Yavaş et al. (2008). For Norwegian and Swedish, we used all child-speech corpora freely available for research. For Swedish, additional spoken and written data produced by adults was selected from open data resources to provide a balanced representation of the language. An overview of this collection is presented in Table 3. For descriptions of the corpora as well as references, see Appendix A.

**Table 2.** Overview of included child-speech corpora, for each of the three languages. All corpora are distributed via CHILDES/PhonBank (MacWhinney, 2000; Rose & MacWhinney, 2014) or listed by the OLAC (Bird & Simons, 2003). For a description of the corpora, see Appendix A.

Language/Dataset		Tokens	Types
Swedish	One corpus; child age 1;0–6;0.	125,147	11,451
Norwegian	Three corpora; child age 1;2–4;1.	73,736	5,495
English	Three British corpora: child age 2;0–7;0.	1,086,386	9,592
	Five American corpora; child age 0;6–8;0.	1,010,545	11,466

**Table 3.** Overview of included Swedish corpora. For a description, see Appendix A.

Language/Dataset	Tokens	Types
Swedish		
Child-directed Speech	290,842	7,241
Adult Speech	2,348,278	85,598
Child-directed Text	350,966	18,840
Adult Text	422,486,506	2,924,922

Concerning the speech data, child-produced and (adult-produced) child-directed data included orthographic transcripts of spontaneous interaction between infants, toddlers, and young children (up to 8 years) and adults in a naturalistic free-play setting at home, or in a lab. These transcripts largely follow the Codes for the Human Analysis of Transcripts (CHAT) format, and are distributed by CHILDES/PhonBank (MacWhinney, 2000; Rose & MacWhinney, 2014) or listed by Open Language Archives Community (OLAC) (Bird & Simons, 2003). The (adult-produced) adult-directed Swedish speech data consisted of orthographic transcripts of spontaneous dialog obtained in a lab or an interview setting. For all speech data, orthographic transcripts were used in their original forms, without special considerations paid to potential variation regarding how non-standard speech forms were represented in the transcripts.

As for written data, the child-directed data encompassed fiction with a child target audience (6–12 years). The adult-directed written data was selected to represent a broad set of genres and domains, consisting of about 25% fiction and 75% informative prose (e.g., news text, official documents, academic texts, periodicals). A detailed description of the sources for the Swedish data set can be found in Appendix A.

### 2.2 Procedure

Lexicon look-up and grapheme-to-phoneme conversion were combined to generate phonemic transcripts (in SAMPA, see https://www.phon.ucl.ac.uk/home/sampa/)^2^ from the orthographic transcripts retrieved from the included corpora. This conversion was performed on unigram frequency lists extracted from the corpora, with words occurring only once in each corpus (i.e., hapax legomena) excluded. For the first step—the lexicon look-up—we used the Swedish NST Lexicon consisting of about 630,000 entries (Andersen, 2005), the Norwegian NST Lexical Database for Norwegian-Bokmål consisting of 753,000 entries (Andersen, 2005), and the English CMU Pronouncing Dictionary (The Carnegie Mellon Speech Group, 2018) consisting of 134,000 entries with North American pronunciation. For words not found in the lexica, phonemic transcriptions were generated using the Sequitur grapheme-to-phoneme (g2p) converter, an open-source tool based on the method described in Bisani and Ney (2008). This way, phonemic transcriptions were generated for (assumed) misspellings (e.g., English: “lotsof” /ˈlɑːt.sɔːf/, “ruldolph” /ˈrʌl.dɑːlf/), non-standard speech forms (e.g., English: “eated” /ˈiːtəd/, Swedish: “dää” /dɛː/, Norwegian: “bæssemor” /^2^bɛ.sə.ˌmuːr/) and otherwise infrequent words (e.g., English: “dimitra” /dɪ.ˈmiː.trə/, Swedish: “startsprej” /^2^stɑːʈ.ˌʂprɛj/, Norwegian: “appelsinjustørk” /a.pəl.^2^siːn.ˌjʉːs.ˌtœrk/) occurring in the corpora. The performance of this method has been tested for Swedish; Strömbergsson et al. (2017) found the reliability of the generated phonemic transcripts to be satisfactory, with a point-by-point agreement with manual phonemic transcriptions of 94%.

As the CMU dictionary includes examples of all major categories of American and British spelling variants (e.g., “-or”/ “-our” or “-ize”/” “-ise”), this procedure also includes normalization between British and American English spelling conventions. That is, common words spelled according to British conventions are transformed into American English pronunciation during lexicon look-up, and any word not found in the lexicon is handled by the g2p model, which has been trained to handle both American and British spelling variants as found in the CMU dictionary.

Speech error patterns were simulated in the phonemic transcripts by automatically replacing specific phonemes or phoneme sequences with other phonemes, across all word tokens in an entire corpus subset. The error patterns were applied one at a time, thus generating one “misarticulated” corpus subset version per error pattern. Table 4 lists the six specific error patterns implemented, selected on the basis of having been frequently attested in at least two of the three included languages. Another selection criterion was practicability—only context-independent error patterns were selected, based on their straightforward implementation. (This excluded context-dependent patterns like metatheses and assimilation.) Furthermore, the simulated patterns were restricted to substitutions with segments that hold phonemic status in all three languages. (This excluded, for example, lateralization of /s/, or an /r/-weakening pattern where /r/ is realized as [w].) One can note that one of the six error patterns—backing—is categorized as atypical in all three included languages (Hua & Dodd, 2006b; Lohmander et al., 2013; Nettelbladt, 2007; 2013), whereas the remaining five are often observed early in typical speech acquisition (Hua & Dodd, 2006b; Lohmander et al., 2013; Nettelbladt, 2007).^3^ Including an atypical error pattern among those investigated allowed the chance of exploring the hypothesis that atypical error patterns have more detrimental effects than typical error patterns.

**Table 4.** The six implemented speech error patterns, together with a description of the operationalization of the implementation, and examples of reported observations illustrating the speech error pattern, for English (EN), Swedish (SW), and Norwegian (NO), respectively. For a more detailed description of the implementation, together with references to where the error patterns have been attested, see Appendix B.

Error pattern	Implementation	Examples
*Segmental patterns*		
(velar) fronting	/k/ → /t/ /g/ → /d/ /ŋ/ → /n/	EN: [ɛd] *egg*, [ti] *key*, [bat] *back*, [tɪn] *king*, [doʊ] *go* SW: [^2^tɑ:ta] *kaka*, [dʉ:l] *gul*, [^2^dɵna] *gunga* NO: [tɔp] *kopp*, [tɑn] *kan*
(coronal) backing	/t, ʈ/ → /k/ /d, ɖ/ → /g/ /n, ɳ/ → /ŋ/	EN: [ku] *two*, [ˈpʌgəl̩] *puddle*, [ˈbɔkəl̩] *bottle* SW: [^1^kɛŋɡə] *tänder*, [^2^maka] *matta* NO: [^2^ø:ɡəˌleɡə] *ødelegge*
stopping	/f/ → /p/ /v/ → /b/ /s, ç, ʃ, θ, tʃ/ → /t/ /ð, z, ʒ, dʒ/ → /d/ /ʂ/ → /ʈ/ /ɧ/ → /k/	EN: [tɛə] *chair*, [peɪs] *face*, [tʌk] *suck*, [tʌm] *thumb* SW: [^1^batən] *vatten*, [^1^to:vər] *sover*, [kɔʈ] *kors* NO: [hʉ:t] *hus*, [^2^li:tə] *lise*
/r/-weakening	/r/ → /j/	EN: [jɛd] *red*, [ˈbɒjoʊ] *borrow* SW: [^2^jamla] *ramla*, [^2^bɑ:ja] *bara*, [døj] *dörr* NO: [jø:] *rød*, [’bæjɛ] *bærer*
*Structural patterns*		
omission of pre-tonic syllable	(C*VC*) + ’C*VC* → ’C*VC*	EN: [ˈbɛlʌ] *umbrella*, [ˈnænə] *banana* SW: [ʹtɑ:tɪs] *potatis*, [^2^le:vəˌtɛj] *leverpastej* NO: [kɛt] *rakett*, [^2^vasceʹsi:nɛn] *vaskemaskinen*
cluster reduction	C_1_C_2_ → C_1_ or C_1_C_2_ → C_2_ C_1_C_2_C_3_ → C_2_	EN: [bɛd] *bread*, [peɪ] *play*, [fɔɡ] *frog*, [faɪ] *fly*, [taɪs] *twice*, [tap] *stop*, [neɪl] *snail*, [saɪd] *slide*, [tit] *street*, [pæʃ] *splash* SW: [^2^kɔka] *klocka*, [^2^fy:sa] *frysa*, [bʉ:n] *brun*, [^1^plɔstɛr] *plåster*, [kɛl] *kväll*, [ki:v] *kniv*, [tu:l] *stol*, [nø:] *snö*, [lɔs] *slåss*, [vans] *svans*, [^2^kata] *skratta* NO: [^2^kipə] *klippe*, [^2^bɪlɛ] *briller*, [^2^tumɛ] *trumme*, [ci:v] *kniv*, [ne:] *sne*, [tu:ɭ] *stol*, [^2^vɑle] *svale*, [vaʈ] *svart*, [^2^ɭaŋə] *slange*

Reported observations of the realization of the six different error patterns have varied. For example, descriptions of cluster reduction illustrate that its realization varies extensively, making implementation particularly challenging (see discussion in McLeod et al., 1997). Further, descriptions of cluster reduction exist primarily for word-initial clusters (Hodson & Paden, 1983; Rose & Inkelas, 2011). In the present study, cluster reduction was not restricted to word-initial position, but was applied in syllable-initial position, thus applying to words like (the Norwegian) “trekker” (English: “pulls”), but also to compounds like (the Norwegian) “juletreet” (English: “the Christmas tree”). This reflects observations reported for both Swedish and Norwegian, and is therefore the desired behavior. For a more detailed description of the implementation of error patterns, together with listed references attesting their realization, see Appendix B. All scripts were computed in Python and are available at https://sprakbanken.speech.kth.se/data/simulerror/.

### 2.3 Clinical survey

A clinical survey was conducted among the audience of a research seminar, held by the first author at an SLP clinic in the Stockholm region, directed to the practicing SLP clinicians employed at this clinic. In all, around 60 individuals participated in this seminar. Forms were distributed to the participants, where the six error patterns (see Table 4) were listed, together with examples illustrating each of them. The participants were instructed to rank the listed error patterns from the least to the most severe, in terms of effects on intelligibility. The rankings were indicated with numbers, ranging from 1 (least severe) to 6 (most severe). On the same form, they were asked to fill in for how long they had practiced as an SLP (in years), and for how long they had worked clinically with children (in years). Forty-seven forms were returned. Fourteen forms were excluded from the analysis; three because they contained ambiguous information, and 11 because the participants had less than one year’s experience of clinical pediatric SLP practice. The remaining 33 forms were included in the analysis, representing participants with an average number of years as a practicing SLP of 7.6 years (max = 38; min = 1; SD = 7.1 years), and an average number of years working with children of 4.8 years (max = 20; min = 1; SD = 3.7 years).

### 2.4 Data analyses

Table 5 lists the five measures used in quantifying the phonological effects of the simulated error patterns. Structural measures were used to characterize the words in the datasets in terms of phonological complexity, in their original version as well as in “misarticulated” versions, thus enabling estimation of the structural impact of “misarticulation.” For example, the difference between the IPC score of the original phonemic transcription of a word, and the IPC score of same word having undergone velar fronting is used as a quantification of the effect of velar fronting for that word type. As an illustration, the Swedish original transcription of the word “gubbar” (Eng. “old men”) /^2^ɡɵ.bar/ yields an IPC score of 1, whereas the velar fronted version of the word, /^2^dɵ.bar/, receives an IPC score of 0; hence, in this case, the velar fronting results in a reduction of complexity by one IPC point. “Misarticulation” resulting in a reduction of complexity would, then, result in negative values, whereas positive values would reflect increased complexity. Relational measures were used to quantify the difference (or “distance”) between original versions and “misarticulated” versions of datasets.

**Table 5.** Structural, relational, and combined measures used for quantifying linguistic structure and/or effects of the simulated speech error patterns, together with a description of the calculation of each measure. As an illustration, calculations and outcome scores are provided of an original phonemic transcription Trsc_orig /^2^spøː.ket/ having undergone stopping, resulting in the “misarticulated” phonemic transcription Trsc_error /^2^pøː.ket/ (Swedish: “spöket,” Eng. “the ghost”).

Measure		General description	Example calculation	Score
*Structural measures*				
IPC	Index of Phonetic Complexity (Jakielski, 1998; Jakielski, 2016)	IPC*_Trsc_error_ –* IPC_*Trsc_orig*_	IPC_*Trsc_orig*_ = 3 (1 for the fricative + 1 for the cluster + 1 for the cluster being heterorganic) IPC_*Trsc_error*_ = 1 (for /p/ and /k/ being heterorganic)	-2
WCM	Word Complexity Measure (Marklund et al., 2018; Stoel-Gammon, 2010).^1.^	WCM*_Trsc_error_ –* WCM_*Trsc_orig*_	WCM_*Trsc_orig*_ = 2 (1 for the fricative + 1 for the consonant cluster) WCM_*Trsc_error*_ = 0	-2
*Relational measures*				
PCC	Percent Consonants Correct (Shriberg & Kwiatkowski, 1982)^2^	The proportion of consonants in PCC_*Trsc_orig*_ that remain unaffected in PCC_*Trsc_error*_.	n consonants in *Trsc_orig* = 4 n unaffected consonants in *Trsc_error* = 3	75%
LVN	Levenshtein distance (Gooskens & Heeringa, 2004; Wieling et al., 2009)	The minimal number of string operations (insertions, deletions or substitutions) required to change *Trsc_orig* into *Trsc_error*. Normalized with regards to string length.	n string operations required = 1 (deletion of /s/) n characters in *Trsc_orig* = 10^3^ n characters in *Trsc_error* = 9 LVN = 1/(10+9)	0.053
*Combined measures*				
PWP	Proportion of Whole-Word Proximity (Ingram, 2002)	pMLU_*Trsc_error*_ / pMLU_*Trsc_orig*_ pMLU = the sum of a) n segments in pMLU_*Trsc_error*_, and b) n correct consonants in pMLU_*Trsc_error*_.	pMLU_*Trsc_orig*_ = 10 (6 for n segments + 4 for n consonants) pMLU_*Trsc_error*_ = 8 (5 for n segments + 3 for n correct consonants)	0.8

1 As no adaptation of the WCM is available for Norwegian, the Swedish adaptation was used also for Norwegian.

2 Here, based on the number of consonants in an original transcription of a word, and the number of consonants left unchanged in the “misarticulated” version of that transcription.

3 Note that the SAMPA version of /^2^spøː.ket/ is /““sp2:$ket/, that is, the marker of accent 2 is indicated by two characters, in contrast to the IPA transcription. Hence, the total number of characters in /””sp2:$ket/ is 10.

All structural measures were calculated on open-class words, that is, excluding entries appearing in pre-specified lists of closed-class words. For the PWP, this follows Ingram’s (2002) original description of the pMLU calculation. For the IPC and the WCM, the original descriptions are unspecified regarding what types of words are included; however, restricting application to open-class words follows previous work of, for example, Howell and Au-Yeung (2007). Lists of closed-class words were created by manual identification of closed-class words, including various forms of auxiliary and copula verbs among the 100 most frequent word types across all corpus subsets. The purely relational measures—PCC and LVN—were calculated on all word tokens, that is, including both closed-class and open-class words. This is in accordance with the original description of the PCC (Shriberg & Kwiatkowski, 1982). Effects of a specific error pattern within a particular corpus subset were expressed as means and standard deviations across word tokens. All scripts analyzing phonological effects were computed in Python and are available at https://sprakbanken.speech.kth.se/data/simulerror/.

The face validity of the rankings provided by each of the five different measures of phonological effects was examined through a Kendall’s τ rank correlation analysis in relation to average intuitive rankings as obtained via the clinical survey. For this analysis, effect scores as obtained by the structural measures of complexity—the IPC and the WCM—raw values were used in the correlation analysis, thus representing difference in complexity regardless of whether the difference resulted in increasing or decreasing complexity.

## 3 Results

### 3.1 Phonological severity ranking: Cross-linguistic variation

**Figure 1.**
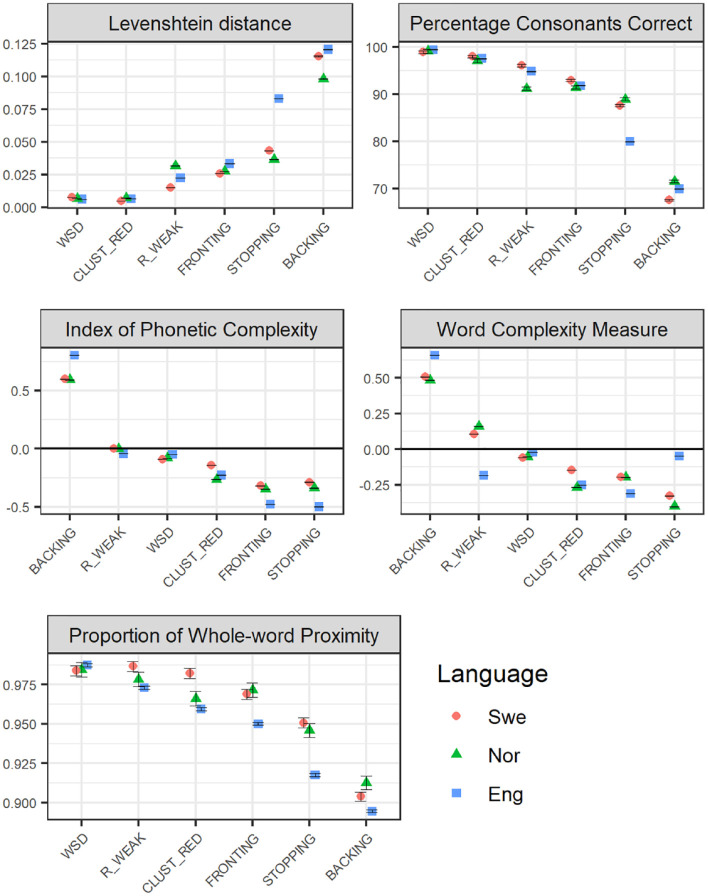
Phonological severity rankings of the six included speech error patterns (WSD: WEAK SYLLABLE DELETION, BACKING, STOPPING, FRONTING, CLUST_RED: CLUSTER REDUCTION, and R_WEAK: /R/-WEAKENING), applied to child-speech datasets across the three languages Swedish (Swe), Norwegian (Nor) and English (Eng), with regard to relational measures (Levenshtein distance, LVN, and the Percentage of Consonants Correct, PCC; top panel), and structural measures (IPC), and Word Complexity Measure, WCM; middle panel), and a measure combining structural and relational features (the Proportion of Whole-word Proximity, PWP; bottom panel). The figures present average values, with error bars representing standard error of the mean. Relational measures (top panel) represent estimations of the distance (LVN) or the accuracy (PCC) that each error pattern results in; smaller effects are represented as lower LVN scores and higher PCC scores. Structural measures (IPC/WCM; middle panel) represent estimations of the reduction (negative values) or increase (positive values) in complexity that the error pattern causes. For the combined measure PWP, smaller effects are represented by higher scores.

Figure 1 illustrates the ranking of the six included error patterns, with regard to phonological severity as measured by relational (top panel), structural (middle panel), and combined measures (bottom panel), based on child-produced Swedish, Norwegian, and English speech. The graphs illustrate a general trend with backing consistently being ranked as having the most severe phonological effects, across all measures, and across all three languages. Another general pattern is that stopping is ranked as the error pattern having the second most severe effects, often larger for English than for the two Scandinavian languages. It can also be noted that, with respect to both structural measures, backing involves an increase in phonological complexity. This is also found for /r/-weakening, but only for Swedish and Norwegian, and only with respect to the WCM; for the IPC, /r/-weakening involves no or a negligible reduction of complexity.

The results concerning the other end of the scale, regarding which of the six error patterns results in the smallest phonological effects, are more divergent. weak syllable deletion is, however, consistently ranked as one of the error patterns resulting in the smallest phonological effects, across the three languages. /r/-weakening is also most often ranked as one of the patterns resulting in the smallest phonological effects.

In terms of differences and similarities across the three languages, the expected difference between English and the two Scandinavian languages is modest. stopping can be identified as the error pattern which exhibits the most salient difference; here, the phonological effects are consistently more detrimental in English than in the two Scandinavian languages. A similar tendency can be observed for backing, where effects are more detrimental in English compared to Swedish and Norwegian; however, this only holds true with regard to the structural and combined measures. More unexpected is the observation of a difference between Swedish on the one hand and Norwegian and English on the other hand, concerning cluster reduction, where effects are more detrimental in Swedish, at least with respect to structural and combined measures.

### 3.2 Phonological severity ranking: Alternative data sources

#### 3.2.1 Age of the speaker

**Figure 2.**
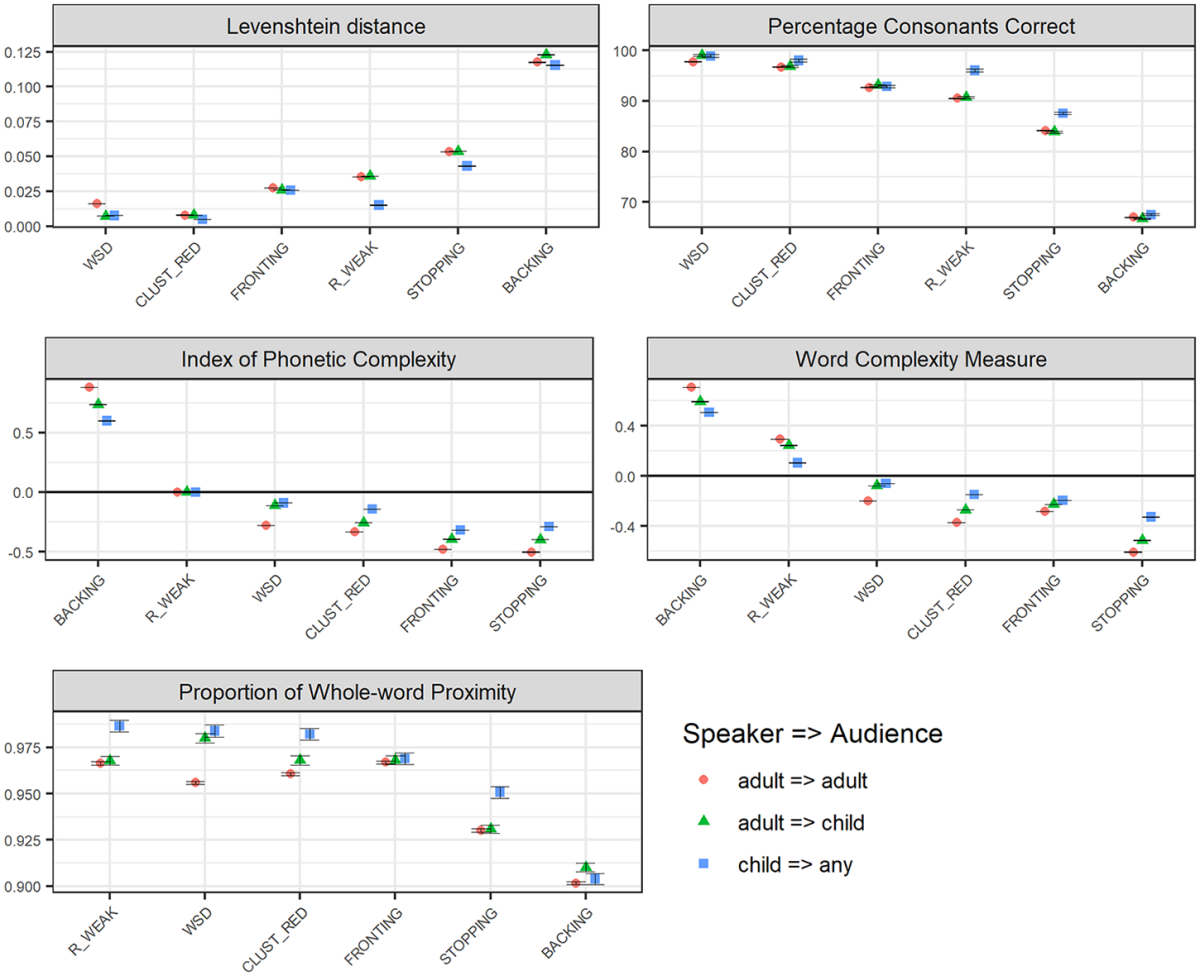
Phonological severity rankings of the six included speech error patterns (WSD: WEAK SYLLABLE DELETION, BACKING, STOPPING, FRONTING, CLUST_RED: CLUSTER REDUCTION, and R_WEAK: /R/-WEAKENING) across Swedish adult-produced speech across the age of the intended audience (child vs. adult), with regard to relational measures (Levenshtein distance, LVN, and the Percentage of Consonants Correct, PCC; top panel), with regard to structural measures (IPC), and Word Complexity Measure, WCM; middle panel), and a measure combining structural and relational features (the Proportion of Whole-word Proximity, PWP; bottom panel). Child-produced speech data is presented as a reference to which the graphs are sorted. The figures present average values, with error bars representing standard error of the mean. Relational measures (top panel) represent estimations of the distance (LVN) or the accuracy (PCC) that each error pattern results in; smaller effects are represented as lower LVN scores and higher PCC scores. Structural measures (IPC/WCM; middle panel) represent estimations of the reduction (negative values) or increase (positive values) in complexity that the error pattern causes. For the combined measure PWP, smaller effects are represented by higher scores.

Figure 2 illustrates that phonological effects of the six included error patterns are often larger when estimated on Swedish adult-produced speech (directed to children), compared to when estimated on child-produced speech extracted from the same conversational setting. However, the difference is generally one of degree rather than of quality—the rank order generally remains the same across the two datasets. (An exception is the ordering of cluster reduction and fronting with regards to the WCM.) Hence, backing results in the largest effects, and weak syllable deletion in the smallest effects, regardless of whether speakers are children or adults. Further, the difference between datasets is smaller with respect to relative measures, compared to when measured by structural and/or combined measures. For the relative measures (PCC and LVN), differences between child-produced and adult-produced speech are subtle, except for /r/-weakening and stopping, where phonological effects are larger in adult-produced speech than in child-produced speech. (For these error patterns, the difference between child-produced and adult-produced speech is also observable in the structural and combined measures.)

#### 3.2.2 Age of intended audience

The variation in phonological effects across the age of the intended audience, that is, whether adult speakers address children or adults, is also illustrated in Figure 2. Here, differences between (adult-produced) child-directed and adult-directed speech are often smaller than differences observed between child-produced and adult-produced speech. Where differences between child-directed and adult-directed speech do occur, it is in the direction of larger effects in adult-directed compared to child-directed speech. The most salient difference is observed for weak syllable deletion; this is the one error pattern where the difference between child-directed and adult-directed speech is consistent across measures (although quite subtly with regards to the PCC). One can also note that for /r/-weakening and stopping, phonological effects do not vary across the age of the intended audience. Instead, a larger difference is observed between the adult-produced speech and the child-produced speech reference, such that effects are more severe in adult-produced speech. Again, the ordering of the error patterns with respect to phonological effects remains largely the same across conditions; hence, backing consistently results in the largest effects in both adult-directed and child-directed speech.

#### 3.2.3 Mode of discourse—spoken vs. written

**Figure 3.**
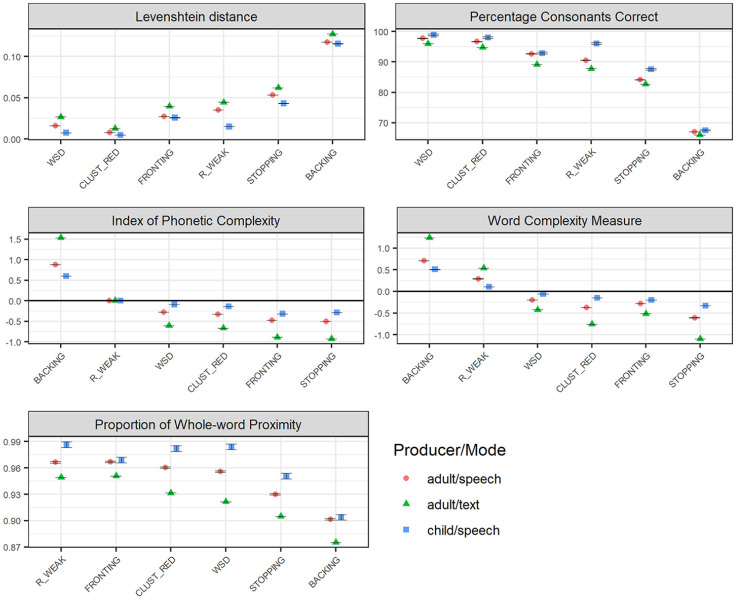
Phonological severity rankings of the six included speech error patterns (WSD: WEAK SYLLABLE DELETION, BACKING, STOPPING, FRONTING, CLUST_RED: CLUSTER REDUCTION, and R_WEAK: /R/-WEAKENING) across Swedish adult-produced speech and text, with regard to relational measures (Levenshtein distance, LVN, and the Percentage of Consonants Correct, PCC; top panel), with regard to structural measures (IPC), and Word Complexity Measure, WCM; middle panel), and a measure combining structural and relational features (the Proportion of Whole-word Proximity, PWP; bottom panel). Child-produced speech data is presented as a reference, to which the graphs are sorted. The figures present average values, with error bars representing standard error of the mean. Relational measures (top panel) represent estimations of the distance (LVN) or the accuracy (PCC) that each error pattern results in; smaller effects are represented as lower LVN scores and higher PCC scores. Structural measures (IPC/WCM; middle panel) represent estimations of the reduction (negative values) or increase (positive values) in complexity that the error pattern causes. For the combined measure PWP, smaller effects are represented by higher scores.

Figure 3 illustrates variation in phonological effects of the six error patterns across different discourse modes: spoken vs. written data. (Note that in order to isolate the modes of the data sources, text vs. speech, only adult-produced data was included in this analysis, as—for natural reasons—very little data was available from children producing text. Child-produced speech data is, however, included for reference.) As the graphs illustrate, basing the analysis on general text data results in larger effects than basing the analysis on speech data produced by adults, and even larger when compared to speech produced by children. (Note, though, that for fronting, the difference between adult-produced and child-produced speech is not as pronounced as for the other error patterns.)

Notably, although the differences between text and speech data are salient with respect to structural and combined measures, they are more subtle with regard to the relational measures (PCC and LVN). Concerning the relational measures, one can observe larger effects for /r/-weakening and stopping in adult-produced language (quite) regardless of mode, compared to child-produced speech. Hence, the results illustrated in Figure 2 for /r/-weakening are reflected here, too: in terms of relational outcome measures, /r/-weakening and stopping result in larger differences from expected targets in adult-produced language compared to in child-produced language. Apart from these observations, Figure 3 illustrates a now familiar overall trend concerning the ordering of error patterns: backing is consistently, and by far, ranked as the pattern resulting in the largest effects, followed by stopping and, although less consistently, fronting. On the other side of the scale, weak syllable deletion and (although less consistently) /r/-weakening and cluster reduction result in the smallest effects.

Visual inspection of the different outcome measures across the described comparisons allows some general observations concerning the nature of the different measures. For example, the two complexity measures—the WCM and the IPC—yield different outcomes regarding /r/-weakening, such that according to the WCM, /r/-weakening results in increasing complexity, whereas with respect to the IPC, it does not affect phonological complexity at all.

### 3.3 Face validity: Rank ordering with regard to intelligibility

**Figure 4.**
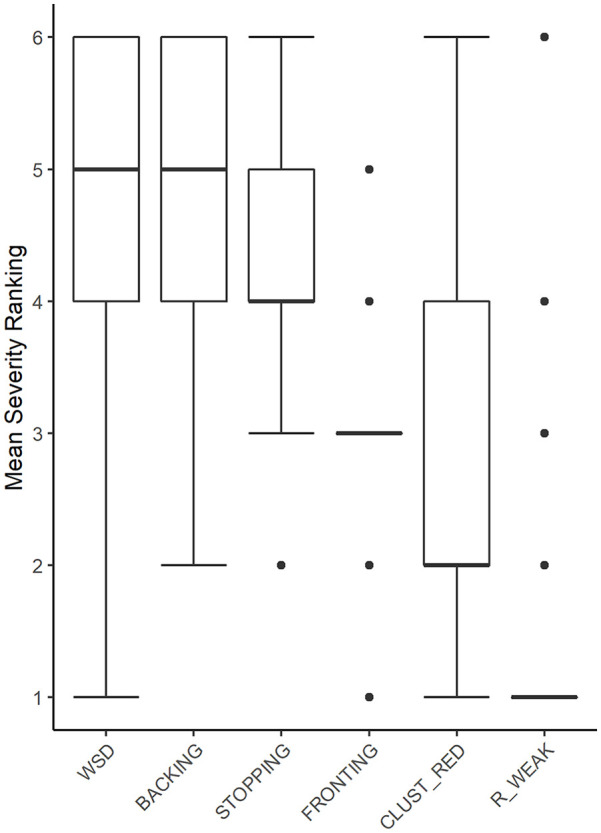
Box-plot diagram showing rankings of the six included speech error patterns (WSD: WEAK SYLLABLE DELETION, BACKING, STOPPING, FRONTING, CLUST_RED: CLUSTER REDUCTION, and R_WEAK: /R/-WEAKENING) with respect to their rated impact on intelligibility, as provided by SLP clinicians (n = 33); 6 = “most severe”, and 1 = “least severe.” Error bars represent standard error of the mean. (For FRONTING and R_WEAK, the interquartile range is 0.)

The results of the ranking of the six error patterns with respect to their impact on intelligibility as provided by Swedish practicing clinicians are presented in Figure 4.

As indicated in these average rankings, /r/-weakening was ranked as having the least detrimental effect on intelligibility. weak syllable deletion was ranked, on average, as having the most detrimental effects on intelligibility, although the distance to backing on the same end of the scale is small. Stopping, fronting and cluster reduction were, on average, ranked as having less severe impact, but still clearly more severe than /r/-weakening.

Table 6 lists the average clinical ranking values together with the average outcome scores of each of the five computed severity measures. Table 7 presents the results of the correlation analyses between the clinically intuitive rankings and the average severity scores as obtained by each of the five included severity measures. (Note that for the two structural measures, the IPC and the WCM, absolute average scores were used in the correlation analyses, rather than the raw values presented in Table 6.) As shown in Table 7, none of the included measures correlate with the intuitive rankings of effects on intelligibility. Furthermore, the table illustrates patterns of interrelations between the five measures, for example, that the PCC and the LVN, that is, the two relational measures, are in close agreement. Also, the WCM correlates strongly with all other measures. The combined measure PWP correlates strongly with the two structural measures, that is, the IPC and the WCM.

**Table 6.** Average values (SD) of the clinically intuitive rankings of the six speech error patterns (WSD: weak syllable deletion, backing, stopping, fronting, CLUST_RED: cluster reduction, and R_WEAK: /r/-weakening) with regard to assumed effects on intelligibility, together with outcomes computed based on the measures Word Complexity Measure (WCM), the (IPC), the Percentage Consonants Correct (PCC), the Levenshtein distance (LVN), and the Proportion of Whole-word Proximity (PWP). All five computed measures are based on child-produced speech data.

Error pattern	Clinical ranking	WCM	IPC	PCC	LVN	PWP
wsd	4.85 (1.5)	-.06 (.36)	-.09 (.55)	99% (7%)	.01 (.05)	.98 (.09)
backing	4.70 (1.3)	.51 (.68)	.60 (.87)	68% (39%)	.12 (.14)	.90 (.13)
stopping	4.18 (1.1)	-.33 (.70)	-.29 (.58)	88% (27%)	.04 (.09)	.95 (.10)
fronting	3.03 (.80)	-.20 (.45)	-.32 (.76)	93% (19%)	.03 (.07)	.97 (.08)
clust_reduct	2.88 (1.3)	-.15 (.45)	-.14 (.56)	98% (8%)	.00 (.02)	.98 (.07)
r_weak	1.36 (1.0)	.11 (.34)	.00 (.00)	96% (13%)	.02 (.05)	.99 (.04)

* *p* < .05.

**Table 7.** Kendall τ correlation coefficients (with p-values, two-tailed) for associations between clinically intuitive rankings of severity with regards to effects on intelligibility and outcomes as measured by each of the included measures of phonological effects (based on child-produced speech data). For the structural measures—the Word Complexity Measure (WCM) and the (IPC)—correlations with clinical rankings are based on absolute raw average scores. For the relational measures—the Percentage of Consonants Correct (PCC) and the Levenshtein distance (LVN)— and for the combined measure Proportion of Whole-word Proximity (PWP), correlational analyses are based on raw average scores.

Measure	Clinical intuition	WCM	IPC	PCC	LVN	PWP
WCM	.33 (.35)	1.00 (–)*				
IPC	.33 (.35)	.73 (.04)*	1.00 (–)*			
PCC	-.20 (.57)	.87 (.02)*	-.60 (.09)*	1.00 (–)*		
LVN	.33 (.35)	.73 (.04)*	.47 (.19)*	-.87 (.02)*	1.00 (–)	
PWP	-.55 (.13)	-.83 (.02)*	-.83 (.02)*	.69 (.06)*	-.69 (.06)	1.00 (–)

* *p* < .05.

## 4 Discussion

This investigation set out to explore phonological effects of six frequently reported speech error patterns when simulated in transcriptions of authentic samples of children’s speech production, and how the effect ranking of these patterns varies across languages, and across different types of linguistic data. We aimed at finding answers to whether findings in one language can be generalized to other languages, and to whether these kinds of investigations of children’s speech acquisition need to rely on the most ecologically valid kind of data (i.e., child-produced speech), or whether they can be based on more readily available data (like, for example, text produced by adult writers). Although replication and extension is necessary to allow definite conclusions, we discuss the knowledge gained from our findings, and what theoretical and practical implications these have.

Concerning the first research question—how the six error patterns are ranked by phonological effects across three different languages—the findings are mostly in line with our expectations. Indeed, the one pattern regarded as atypical in the included languages, backing, was consistently ranked as causing the largest phonological effects, across measures, and across the three languages. Almost as consistently, stopping was ranked as the pattern causing second largest effects. Less expected was the finding that the two error patterns involving omission rather than substitution of segments—weak syllable deletion and cluster reduction—were ranked toward the other end of the scale, that is, as causing the smallest phonological effects, across measures, and across the three languages. A possible explanation to this somewhat surprising finding is that the data underlying the analysis—that is, child-produced speech—offer fewer opportunities where these patterns are applicable, compared to language produced by adults. This would align to suggestions of lexical selectivity (e.g., Vihman et al., 2014; Willadsen, 2013), that is, that children’s speech production is skewed by their preference to produce words that are composed of phonological elements and structures that they master. The finding that these patterns have larger phonological effects in adult-produced data corroborates this hypothesis. On the surface, the surprisingly small effects caused by weak syllable deletion and cluster reduction in child-produced speech appear to oppose Hodson and Paden’s (1981) suggestion that omissions are more disruptive than substitutions. However, whereas Hodson and Paden make claims about effects on intelligibility, the present study cannot make claims beyond effects as measured by phonological structure and accuracy; hence, this seeming contradiction may adhere to the fact that phonological effects do not necessarily correspond to level of intelligibility.

In terms of the rank order of severity, there are no dramatic cross-linguistic differences—the six patterns are ranked similarly across the three languages. This can be expected, as all three languages share many typological traits. There were, however, some differences in the size of the phonological effects. For one, stopping was found to have larger effects in English than in the Scandinavian languages, thus confirming the expected pattern of cross-linguistic differences. Examination of the most frequent words in the three respective languages reveals that many of the top-most frequent words in English contain fricatives (e.g., “the,” “this,” “is”), whereas this is not the case for the corresponding words in the Scandinavian languages (e.g., “det,” “den,” “är”). This raises the suspicion that the difference is driven by the high frequency of fricatives in the most frequent English words. However, as closed-class words were excluded from the analysis based on structural and combined measures, this fact alone cannot explain the difference. Rather, the fact that English has more fricatives overall—reflected in both types and tokens—than the Scandinavian languages could be a more plausible explanation. Consequently, stopping can be expected to cause more problems in English than in the Scandinavian languages.

Less expected was the observation concerning cluster reduction, where smaller effects were observed for Swedish than for English and Norwegian. Indeed, the proportion of word tokens in each language where cluster reduction was applied was smaller for Swedish (5%) compared to Norwegian (8%) and English (7%). A possible explanation behind this unexpected variation could be that either the Swedish or the Norwegian child-produced speech dataset is skewed. Considering that these corpora consist of data longitudinally collected from only a few children, this is quite possible. By extending the analysis to include other types of linguistic data in English and Norwegian, such tendencies in the child-speech data could be revealed.

Regarding the second research question, concerning the variation in phonological effects across different types of linguistic sources, the results show a general trend of phonological effects being the largest in general text data, followed by adult-produced speech directed to adults and adult-produced speech directed to children, where effects in turn were larger than in child-produced speech. This illustrates that there are indeed differences between the datasets in terms of their phonological characteristics, in line with previous observations (Strömbergsson et al., 2017). However, as the difference is primarily one of degree rather than of quality—the rank order remains largely the same across speaker and listener age—there are aspects in adult-produced speech that can be generalized to child-produced speech. Although this finding needs replication in other languages, it is an indication that large-scale investigations of ranking of severity of different error patterns may be based on adult speech data in cases where authentic child-speech data is not available. In cases where the specific magnitude of the effect is important, this is, however, not recommended.

Concerning adult-produced speech directed to other adults and to children, respectively, and the difference observed with regard to weak syllable deletion, this could be seen as a confirmation of lexical selectivity as an explanation to why this error pattern results in surprisingly small effects in the child-produced speech data. One can note that the difference is primarily one between child-produced speech and adult-produced speech *directed to adults*, rather than one driven by the age of the speaker. This may be a consequence of the adults adapting their speech to the child they are talking to. A similar trend can be seen in the comparison across different modes of discourse (speech vs. text). At least with reference to complexity measures and combined measures, this trend even affects the rank ordering of the error patterns, so that weak syllable deletion has similar or even more degrading effects than fronting.

The patterns /r/-weakening and stopping were found to result in larger phonological effects in adult-produced language (text and speech) than in child-produced speech. Notably, the difference in effect size between adult-produced speech and child-produced speech was larger than that observed between child-directed and adult-directed speech produced by adult speakers. Hence, even though the speech produced by children and most of the child-directed speech produced by adults were retrieved *from the same conversational data*, fricatives and /r/s occur more frequently in the adults’ than in the children’s speech production. In that respect, there were no signs of adult adaptation to the children’s phonological or lexical preferences. It should be cautioned, however, that a possible explanation could be sought in the original orthographic transcripts. Word-final /r/s are often reduced by Swedish speakers of all ages. However, we have found that in the Swedish child-speech corpus used in this study, word-final “r”s are orthographically transcribed in adults’ speech but less so in the orthographic transcripts of children’s speech. An exploratory study of ten frequent Swedish verbs in present tense showed that 57% of all occurrences in the children’s speech are transcribed as the reduced form compared with less than 3% in the adults’ speech. Taken at face value, this suggests that the children’s speech is more reduced than the adult’s speech. However, we wonder if the difference is due to the purpose of the transcripts: the child-speech transcripts describe the performance of the child during speech development, whereas the competence of the adult speaker is taken for granted in the adult transcripts.

The ordering of error patterns as provided by the panel of clinicians closely matches expected phonological development, with the error patterns ranked as having the most detrimental effects on intelligibility being the ones that are typically only seen in the earliest stage of phonological acquisition (or, in the case of backing, not expected at all in typical phonological acquisition). So, for example, weak syllable deletion, ranked by the clinicians as the pattern having the most detrimental effects on intelligibility, is also an error pattern which is expected to be overcome early in speech acquisition (Nettelbladt, 2007), whereas /r/-weakening, ranked as the least detrimental pattern, is observed in typically developing children as late as around seven years of age (Nettelbladt, 2007). It cannot be ruled out that the clinicians’ knowledge and experience of these norms may have influenced their ranking decisions. Regarding the relation between the included severity measures and the clinical intuitive rankings, no correlation was found. Hence, none of the severity measures reaches face validity. As alluded to before, this may be a consequence of the measures being sensitive to broad phonological characteristics, whereas the clinical intuitive rankings were given with reference to effects on intelligibility; maybe, there is simply not a straightforward correspondence between these two aspects of speech production. It is possible that more sensitive phonological measures would better reflect perceptual similarity/distance between target and “misarticulated” transcriptions, such as by weighting phonological similarity between symbols. This would take into account, for example, that vowels are more similar to other vowels than to consonants, and that fricatives are more similar to other fricatives than to other consonants (see e.g., Preston et al., 2011). For measures purporting to reflect functional effects of deviation from expected target forms this is a desirable feature to be implemented in future work. Doubtlessly, there are also non-phonological factors that contribute to intelligibility, such as the linguistic context, to which the analysis in the present study is insensitive. Moreover, factors like functional load, that is, the quantification of the effect of a loss of a phonological contrast in a language (Stokes & Surendran, 2005), can also be assumed to influence intelligibility. Including contextual information as well as lexical factors like functional load are natural and important venues for future extensions to this work.

A couple of reflections regarding the nature of the included severity measures deserve mentioning. First, it can be noted that according to the structural measures, backing and—although to a lesser extent—/r/-weakening involve increasing articulatory complexity. This is a natural consequence of velar speech sounds being categorized by these measures as more complex than alveolar/dental speech sounds (Jakielski, 2016; Marklund et al., 2018; Stoel-Gammon, 2010), which, in turn, is motivated by an expected later age of acquisition of these speech sound types. In other words, if early acquired (i.e., less complex) sounds are replaced by late acquired (i.e., more complex) sounds, this will be reflected as increased complexity. For backing, this adds to the already established view of this pattern as atypical (Dodd, 2005; Hodson & Paden, 1983). For /r/-weakening, the counterintuitive finding that it should lead to unchanged or even increasing complexity (as observed with regards to the WCM) is a consequence of the phonological classification of the /r/ and /j/ sounds in the included languages. In all three languages, /r/ can take many allophonic forms—in Swedish, for example, [ʐ ʂ ɹ ɾ] (Engstrand, 2004). The phonological classification that both the IPC and the WCM are based on requires reducing this variation and selecting one of these allophonic variants as representing /r/. For all three languages, /r/ was classified as a voiced alveolar rhotic sound. For Swedish and Norwegian, /j/ was classified as a voiced palatal fricative. As the Swedish version of the WCM awards /r/ with one point, but voiced fricatives with two points, the substitution of /j/ for /r/ is reflected in increased complexity. In English, /j/ was categorized as a voiced palatal approximant, representing a sound type which is not awarded any complexity points at all; hence the substitution of /j/ for /r/ will result in reduced complexity in English. This illustrates an inherent restriction in allowing one symbol to represent the many allophonic shapes and forms a specific speech sound may take, and consequently, an issue that needs to be supplemented by investigations based on actual speech data.

Another reflection concerning the nature of the severity measures can be made regarding the close correspondence between the Levenshtein distance and the PCC. Although both these measures are based on counts of symbol omission, insertion, and substitution, they are different in respect of whether they measure distance or similarity (hence, they are inverted), and whether all symbols or only consonants are considered. However, for all implemented error patterns except weak syllable deletion, only consonants were affected, and hence, the measures were expected to be closely (inversely) correlated. This observation may motivate researchers to rely on the Levenshtein distance instead of the PCC in large-scale studies of phonological accuracy, as there exist many freely available scripts to use for its implementation, whereas implementation of the PCC may be more cumbersome. In the present study, the PCC was based on the number of consonant changes made to the original transcriptions in the simulation of misarticulation. In studies where the only information that exists is a target transcription and an observed transcription, the calculation of the PCC will have to rely on other procedures.

Concerning the results for English, it should be borne in mind that the results are based on American English pronunciation variants, stemming from the American lexicon from which phonemic transcriptions are retrieved/derived. This is perhaps most relevant to the results concerning /r/-weakening, where British English phonemic transcriptions would have offered fewer opportunities where this pattern would be applicable. For example, in words like “car,” the word-final “r” would be dropped in many British English variants, disallowing /r/-weakening to be applied. Hence, the effects of /r/-weakening presented here can be expected to be more pronounced than they would have been if they were instead based on British English. In this context, it should also be acknowledged that the selected /r/-weakening pattern where /r/ is substituted for [j] is considerably less common in English than /r/ substituted for [w] (Smit, 1993). This serves to underline that the primary value of cross-linguistic comparisons like the present is not the absolute values presented for each of the included languages, but rather, the relation between values across the included languages.

### 4.1 Limitations and future research

In all corpus-based studies, there is a risk that the corpora are not wholly representative of the language discourse the researchers intend to examine. This risk is present also here and isolating only one factor when comparing different languages and data sources proved to be challenging. For example, in the cross-linguistic analysis, there are other factors that vary between the Swedish, Norwegian, and English datasets than merely the language, such as the age of the children included. This should be kept in mind in the interpretation of the findings.

As mentioned above, the included orthographic transcripts do not always follow standard orthographic spelling. For example, in the Swedish CHILDES data, spellings may sometimes reflect both common reductions and rarer misarticulation, like “fö” (for “för,” English: “for”), “fyplan” (for “flygplan,” English: “airplane”). In cases where non-adult-like learner forms like the English “eated” occur in the orthographic transcripts, this is not a problem. Such cases are handled by the grapheme-to-phoneme conversion, which provides a phonologically reasonable guess of how the previously unseen word is produced. However, in cases where the speaker’s reductions/misarticulations are represented in the orthographic transcripts (such as in “fyplan” mentioned above, where a cluster reduction is represented), the estimation of the effect of the simulated misarticulation will be obscured. As the misarticulation is already in the original production, it will—erroneously—be assumed to be the target production. On close examination, this phenomenon was indeed found in the Swedish CHILDES data, as described above. For the English and Norwegian datasets, this was rarer. This should serve to encourage Swedish child-speech researchers to collect and share their data, in order to increase the availability of higher-quality child-speech resources in Swedish. From the authors’ point of view, orthographic documentation of speech data should follow standard spelling conventions, ideally with each orthographic word aligned to a phonemic transcription.

The methodological approach used in this study in the simulation of misarticulation is based on a number of assumptions, each of which may be questioned. For one, the error patterns are implemented across the board in an entire dataset. Obviously, this is not an exact representation of children’s speech production in real-life. For example, although consistent velar fronting has been attested in many cases (e.g., Cleland et al., 2017), a tendency for velar fronting to be driven by word/syllable context has also been reported (McAllister Buyn, 2012; Mason et al., 2015). Variation in speech production driven by phonotactic frequency is also not reflected (see James et al., 2008). Restraining the distribution of error patterns by syllabic/word context and taking phonotactic frequency into account is feasible within the suggested framework. This is an interesting venue for future work.

Regarding the set of quantitative measures implemented in the present study, it should be noted that all were originally designed to be applied to actual samples of children’s speech production. Hence, their values may be conspicuously inflated when applied to thousands, or even millions, of words as in the present study. Typically, in the measurement descriptions, minimum sample sizes are specified (e.g., 100 words for the PCC in Shriberg & Kwiatkowski, 1982), whereas upper limits are rarely considered.

The dataset consistently used as a reference in the present study was Swedish child-produced speech data. No investigation was made into whether results for the other two languages differed dependent on the age of the producer or the audience, or between the modes of the data. This remains a topic for future studies. Also, although there are expected differences between the two Scandinavian languages and English, all three languages included here are from the Germanic language family. Based on previous research observing cross-linguistic differences in phonological structure between children’s speech production in English compared to other languages, such as Finnish and Cantonese (e.g., Hua & Dodd, 2006b; Saaristo-Helin et al., 2006), another interesting focus of future studies would be to extend the investigation to include languages that differ more from English. This would complement important large-scale cross-linguistic investigations like those proposed by Bernhardt and colleagues (2017) and pursued by McLeod and Crowe (2018), in addressing a calling need for increased knowledge into phonological acquisition in a wide range of languages other than English.

## 5 Conclusions

This investigation is explorative in its nature, and—to our knowledge—the first large-scale investigation of phonological consequences of different speech error patterns across languages, different modes, and age of speaker and intended audience. The rank order of speech error patterns remained rather stable across the three included languages, with backing being ranked as the most severe across all three languages. Hence, although backing was already known to be an atypical error pattern, our findings add the information that it also causes more detrimental phonological effects than the other five, thus serving as support to prioritize it in clinical intervention. Further, stopping was found to cause more detrimental effects in English compared to the two Scandinavian languages. This effect may be linked to typological differences between the included languages. The observation that rank order remained rather stable across modes of discourse and speakers’ ages indicates that for the purpose of ordering error patterns by phonological effects, readily available sources like text corpora may be used as a proxy for the ideal and most ecologically valid data. Finally, the finding that none of the included metrics of phonological effects reflected clinicians’ intuitive ratings of different speech error patterns in terms of effects on intelligibility corroborates earlier suggestions that phonological competence does not necessarily translate into level of intelligibility.

## Appendix A

**Table A1.** Overview of included child speech corpora, for each of the three languages. All corpora are distributed via CHILDES/PhonBank (MacWhinney, 2000; Rose & MacWhinney, 2014), except for The Simonsen Corpus; This corpus was distributed by CHILDES on March 11, 2016, and is listed as a Norwegian resource by the Open Language Archives Community (OLAC; Bird & Simons, 2003) as of February 11, 2019.

Language/Dataset		Tokens	Types	Reference
Swedish				
	The Lund corpus. Free play with caregiver at home. 5 children. Longitudinal, from approx. 1 to 6 years old.	125 147	11 451	Strömqvist, Richthoff & Andersson (1993)
	*Complete Swedish child speech dataset*	*125 147*	*11 451*	
Norwegian				
	The Garmann Corpus. Spontaneous parent-child interaction at home. 8 children. Longitudinal, from approx. 1 to 2 years old. Distributed by CHILDES.	11 770	1 079	Garmann, Hansen, Simonsen & Kristoffersen (in press)
	The Ringstad Corpus. Spontaneous parent-child interaction at home. 3 children. Longitudinal, from age 1;10 to 2;8. Distributed by CHILDES.	49 082	4 203	Ringstad (2014)
	The Simonsen Corpus. Spontaneous interaction with an adult. 2 children. Longitudinal, from approx. 2 to 4 years old.	12 884	1 729	Simonsen (1990)
	*Complete Norwegian child speech dataset*	*73 736*	*5 495*	
English				
	The Fletcher Corpus. Interview setting. 72 children: 3, 5 and 7 years old. British English. Distributed by CHILDES.	80 581	2 951	Fletcher & Garman (1988)
	The Thomas Corpus. Free play with caregiver at home. One child. Longitudinal, age 2;0–5;0. British English. Distributed by CHILDES.	486 807	6 865	Lieven, Salomo & Tomasello (2009)
	The Manchester Corpus. Free play with caregiver at home. 12 children. Longitudinal, from approx. 2 to 3 years old. British English. Distributed by CHILDES.	518 998	5 741	Theakston, Lieven, Pine & Rowland (2001)
	The Braunwald Corpus. Parent-child interaction at home. Longitudinal. One child, age 1;5–7;0. American English. Distributed by CHILDES.	56 524	2 689	Braunwald (1978)
	The Brown Corpus. Parent-child interaction at home. 3 children. Longitudinal, from approx. 2 to 5 years of age. American English. Distributed by CHILDES.	283 967	4 831	Brown (1973)
	The Providence Corpus. Spontaneous parent-child interaction at home. 6 children. Longitudinal, from approx. 1 to 3 years of age. American English. Distributed by CHILDES.	333 413	6 796	Demuth, Culbertson & Alter (2006)
	The MacWhinney Corpus. Parent-child interaction at home. 2 brothers. Longitudinal, from age 0;6–8;0 and 0;7–5;6. American English. Distributed by CHILDES.	156 958	5 263	MacWhinney (1991)
	The Weist Corpus. Parent-child interaction at home or in a lab setting. Longitudinal, from approx 2 to 5 years of age. American English. Distributed by CHILDES.	179 683	4 914	Weist, Pawlak & Hoffman (2009)
	*Complete English child speech dataset*	*2 096 931*	*15 712*	

**Table A2.** Description of the Swedish (adult-produced) corpora selected according to linguistic mode (spoken/written) and target audience (children/adults).

Dataset	Corpus description	Tokens	Types	Reference
*Child-Directed Speech*				
	The Lund corpus (distributed by CHILDES). Spontaneous adult-child interaction; home environment; 5 children (approx. age 1;0 to 6;0).	244 412	6 069	Strömqvist, Richthoff & Andersson (1993)
	LONG-MINGLE. Spontaneous parent-child interaction; lab environment; free play scenario, 17 children (approx. age 0;3-2;9)	46 430	2 533	Björkenstam (2014)
	*Total Child-Directed Speech*	*290 842*	*7 241*	
*Adult-Directed Speech*				
	Gothenburg Dialogue Corpus. Spoken interaction; Context-goverened sample. Distributed by Språkbanken, Gothenburg University.	1 345 044	49 907	Allwood, Björnberg, Grönqvist, Ahlsén, & Ottesjö (2000)
	Spontal. Spontaneous speech in a lab environment; 120 dyads.	79 326	6 309	Edlund, Beskow, Elenius, Hellmer, Strömbergsson & House (2010)
	Swedia 2000. Interview transcripts; speakers of Swedish dialects.	923 908	46 896	Eriksson (2004)
	*Total Adult-Directed Speech*	*2 348 278*	*85 598*	
*Child-Directed Text*				
	Fiction. A subset of the corpus LäSBarT (Mühlenbock, 2009) - Lättläst svenska och barnbokstext, consisting of child fiction (sample criterion: target audience age: 6-12 years).	350 966	18 840	Mühlenbock (2009)
	*Total Child-Directed Text*	*350 966*	*18 840*	
*Adult-Directed Text*				
	A combination of corpora distributed by Språkbanken: news text (60.2%), official texts (0.2%), academic text, social sciences (4.6%), academic text, the humanities (4.8%), medical news periodical (5.4%), and fiction (24.8%).	23 894 290	577 568	Corpora: *Göteborgsposten 2013*; *Förvaltningsmyndigheters texter*; A subset of *Akademiska texter - samhällsvetenskap*; A subset of *Akademiska texter – humaniora*; *Läkartidningen 2005*; *Norsteds romaner 1999*; *Bonniers romaner II*. Distributed by Språkbanken, Gothenburg University.
	Stockholm-Umeå Corpus 2.0. A balanced corpus of Swedish.	1 021 606	93 096	Källgren (2006)
	N-grams for Swedish (based on the NST Text Corpus); news text (80%), fiction (6%), periodicals (1.5%), miscellaneous, e.g., web text (13%).	397 570 610	2 683 681	‘N-grams for Swedish (based on NST news text)’, (2012), distributed by Språkbanken, Oslo, Norway: Nasjonalbiblioteket.
	*Total Adult-Directed Text*	*422 486 506*	*2 924 922*	
*TOTAL DATA SET*		*425 601 739*	*2 961 919*	

## Appendix B

**Table B1.** Implemented speech error patterns, together with a description of the operationalization of the implementation, examples of reported observations illustrating the error pattern, for English (EN), Swedish (SW) and Norwegian (NO), respectively. Attested observations come from both young children at early stages of phonological acquisition, and from children with speech sound disorders.

Error pattern	Implementation	Examples	Attested in
(velar) fronting	/k/ → /t/ /ɡ/ → /d/ /ŋ/ → /n/	EN: [ɛd] *egg*, [ti] *key*, [bat] *back*, [tɪn] *king*, [doʊ] *go*	Grunwell (1987); Rose & Inkelas (2011); Hodson & Paden (1983); Yavaş (1998); Bernhardt & Stoel-Gammon (1994); Bernhardt & Stemberger (1998); Ingram (1976); McIntosh & Dodd (2009); Smit (1993a)
		SW: [^2^tɑ:ta] *kaka*, [dʉ:l] *gul*, [tfɛl] *kväll*, [^2^dɵna] *gunga*, [^2^tnapa] *knappar*	Nettelbladt (2007); Magnusson (1983); Eneskär (1978); Nettelbladt (1983)
		NO: [tɔp] *kopp*, [tɑn] *kan*	Simonsen (1990); Vanvik (1971)
(coronal) backing	/t, ʈ/ → /k/ /d, ɖ/ → /ɡ/ /n, ɳ/ → /ŋ/	EN: [ku] *two*, [ˈpʌɡəl̩] *puddle*, [ˈbɔkəl̩] *bottle*, [ˈæŋɡu̩] *handle*	Rose & Inkelas (2011); Hodson & Paden (1983); Smith (1973); Smit (1993a)
		SW: [^2^kɛŋɡə] *tänder*, [^2^maka] *matta*	Nettelbladt (2007); Magnusson (1983)
		NO: [^2^ø:ɡəˌleɡə] *ødelegge*^1^	Vanvik (1971)
stopping	/f/ → /p/ /v/ → /b/ /s, ç, ʃ, θ, tʃ/ → /t/^2^ /ð, z, ʒ, dʒ/ → /d/ /ʂ/ → /ʈ/ /ɧ/ → /k/	EN: [tɛə] *chair*, [tɪp] *ship*, [peɪs] *face*, [tʌk] *suck*, [tʌm] *thumb*	Grunwell (1987); Hodson & Paden (1983); Yavaş (1998); Bernhardt & Stoel-Gammon (1994); Hodson & Paden (1981); Bernhardt & Stemberger (1998); McIntosh & Dodd (2009); Smit (1993a)
		SW: [^1^batən] *vatten*, [^1^to:vər] *sover*, [tɛp] *käpp*, [^2^bɪspa] *vispa*, [kɔʈ] *kors*, [kɑ:] *ska*, [nɑ:ʈ] *snart*, [bo:r] *svår*, [tmɵt] *smuts*	Nettelbladt (2007); Magnusson (1983); Linell & Jennische (1980)
		NO: [hʉ:t] *hus*, [^2^li:tə] *lise*, [^2^tɭucə] *slukke*	Vanvik (1971); Simonsen (1990)
/r/-weakening	/r/ → /j/	EN: [jɛd] *red*, [ˈbɒjoʊ] *borrow*	Bernhardt & Stemberger (1998); Hewlett (1992); Smit (1993a)
		SW: [^2^jamla] *ramla*, [^2^bɑ:ja] *bara*, [døj] *dörr*	Netin & Pehrson (2014); Magnusson (1983); Nettelbladt (1983); Linell & Jennische (1980)
		NO: [jø:] *rød*, [^1^bæjɛ] *bærer*	Simonsen (1990); Lindsjørn & Vethe (2013); Fintoft et al. (1983)
weak syllable deletion	(C*VC*)+’C*VC* → ’C*VC* *For Swedish and Norwegian: delete non-stressed syllables between syllables with main and secondary stress:* ˈS_1_S_2_(S+)ˌS_2_ → ˈS_1_S_2_ˌS_3_	EN: [ˈbɛlʌ] *umbrella*, [ˈnænə]/[ˈnɑnə] *banana*	Grunwell (1987); Velleman & Shriberg (1999); Rose & Inkelas (2011); Hodson & Paden (1983); Ingram (1976); Yavaş (1998); Gerken & McGregor (1998); McIntosh & Dodd (2009); Masso et al. (2016)
		SW: [^1^tɑ:tɪs] *potatis*, [^2^le:vəˌtɛj] *leverpastej*, [di:n] *gardin*	Nettelbladt (2007); Netin & Pehrson (2014); Magnusson (1983); Linell & Jennische (1980)
		NO: [kɛt] *rakett*, [^1^tɑ:ɭɛ] *betale*, [^2^vasceʹsi:nɛn] *vaskemaskinen*, [pi:r] *papir*	Simonsen (1990:172); Vanvik (1971)
cluster reduction			
obstruent + approximant^4^	C_1_C_2_ → C_1_	EN: [bɛd] *bread*, [peɪ] *play*, [klɑk] *clock*, [fɔɡ] *frog*, [faɪ] *fly*, [taɪs] *twice*	Grunwell (1987); Yavaş (1998); McIntosh & Dodd (2009); Ingram (1976); Klopfenstein & Ball (2010); Hewlett (1992); Smit (1993b)
		SW: [^2^kɔka] *klocka*, [^2^fy:sa] *frysa*, [bʉ:n] *brun*, [^1^plɔstɛr] *plåster*	Magnusson (1983); Nettelbladt (2007); Nettelbladt (1983); Eneskär (1978)
		NO: [^2^kipə] *klippe*, [^2^bɪlɛ] *briller*, [^2^tumɛ] *trumme*	Simonsen (1990); Vanvik (1971)
stop + /v|nasal/	C_1_C_2_ → C_1_	EN: -	
		SW: [kɛl] *kväll*, [to:] *två*, [ki:v] *kniv*	Linell & Jennische (1980); Eneskär (1978); Bjar (2010)
		NO: [ci:v] *kniv*^3^	Simonsen (1990)
/s/ + (stop|nasal)	C_1_C_2_ → C_2_	EN: [tap] *stop*, [neɪl] *snail*, [maɪl] *smile*	Grunwell (1987); Yavaş & McLeod (2009); Ingram (1976); Yavaş (1998); Smit (1993b)
		SW: [tu:l] *stol*, [pi:s] *spis*, [nø:] *snö*	Magnusson (1983); Eneskär (1978); Linell & Jennishe (1980)
		NO: [ne:] *sne*, [muk] *smukk*, [tu:ɭ] *stol*, [kɑ] *skall*	Simonsen (1990); Kristoffersen & Simonsen (2006); Vanvik (1971); Lindsjørn & Vethe (2013)
/s/ + any other consonant	C_1_C_2_ → C_2_	EN: -	
		SW: [lɔs] *slåss*, [vans] *svans*	Nettelbladt (1983); Eneskär (1978); Magnusson (1983)
		NO: [^2^vɑle] *svale*, [vaʈ] *svart*, [^2^ɭaŋə] *slange*	Kristoffersen & Simonsen (2006); Lindsjørn & Vethe (2013)
/s/ + approximant	C_1_C_2_ → C_1_	EN: [sɪk] *slick*, [saɪd] *slide*	Yavaş & McLeod (2009); Yavaş (1998: 136); Yavaş et al. (2008); Grunwell (1987); Klopfenstein & Ball (2010); Smit (1993b)^5^
	
	
/s/ + Plosive + Approximant	C_1_C_2_C_3_ → C_2_	EN: [tit] *street*, [pæʃ] *splash*	Grunwell (1987); Yavaş (1998); Smit (1993b)
		SW: [^2^kata] *skratta*	Magnusson (1983)
		NO: N/A^6^	

1 To our knowledge, this is the only published observation of an example of a possible case of coronal backing in Norwegian. Note, however, that the example may well be contextually driven, i.e. an assimilation.

2 In cases of /sC/-clusters, the /s/ is removed.

3 No reports found of the form stop+nasal. Now treated as in Swedish, i.e. reduction to stop, except when C_2_ is syllabic. (*børsten* -> /^2^bøʈɳ̍/)

4 Except for /sl/ in English, which is treated separately.

5 But see Hewlett (1992) and Ingram (1976) for reports of omission of C_1_, and Yavaş (1998: 136) and Klopfenstein and Ball (2010) for reports on omission of either C_1_ or C_2_.

6 We have not found any reports of observed reductions of /sCC/ clusters in Norwegian.

## Notes

1. We use the term “error pattern,” to describe children’s systematic deviations from expected target forms, often realized as perceived substitutions or omissions of speech sounds. We prefer this term over the term “phonological process,” to avoid suggesting claims regarding the cause of the observed pattern (see Hua & Dodd, 2006a and Rvachew & Brosseau-Lapré, 2012, among others).

2. Although all phonemic transcriptions were in SAMPA, we indicate phonemic transcriptions throughout the manuscript in IPA, for better readability.

3. Note, however, that backing is a developmentally typical error pattern in some languages, such as Cantonese (To et al., 2013) and Vietnamese (Phạm & McLeod, 2019).
